# Long Non-Coding RNA THRIL Promotes Influenza Virus Replication by Inhibiting the Antiviral Innate Immune Response

**DOI:** 10.3390/v17020153

**Published:** 2025-01-23

**Authors:** Mengying Chen, Jingyun Hu, Xinni Zhou, Ming Gao, Ning Li, Guihong Yang, Xiaojuan Chi, Song Wang

**Affiliations:** 1 Key Laboratory of Animal Pathogen Infection and Immunology of Fujian Province, College of Animal Sciences, Fujian Agriculture and Forestry University, Fuzhou 350002, China; 2 Key Laboratory of Fujian-Taiwan Animal Pathogen Biology, College of Animal Sciences, Fujian Agriculture and Forestry University, Fuzhou 350002, China

**Keywords:** long non-coding RNAs, THRIL, influenza A virus, innate immunity, IRF3

## Abstract

Long non-coding RNAs (lncRNAs) have been recognized for their crucial roles in the replication processes of various viruses. However, the specific functions and regulatory mechanisms of many lncRNAs in influenza A virus (IAV) pathogenesis remain poorly understood. In this study, we identified lncRNA THRIL and observed a significant reduction in its expression following IAV infection in A549 cells. The treatment of cells with the viral mimic poly (I:C), or with type I and type III interferons, resulted in a substantial decrease in THRIL expression. Furthermore, THRIL overexpression significantly enhanced IAV replication, while its silencing markedly reduced IAV replication. Additionally, IAV infection led to notable reductions in the expression levels of type I and type III interferons in cell lines overexpressing THRIL compared to control groups; conversely, cell lines with THRIL knockdown exhibited significantly higher interferon levels than control groups. Moreover, THRIL was found to inhibit the expression of several critical interferon-stimulated genes (ISGs), which are essential for an effective antiviral response. Notably, our findings demonstrated that THRIL impaired the activation of IRF3, a key transcription factor in the interferon signaling pathway, thereby suppressing host innate immunity. These results highlight THRIL’s potential as a therapeutic target for antiviral strategies.

## 1. Introduction

The influenza virus is taxonomically classified within the family Orthomyxoviridae as an RNA virus that is responsible for causing influenza in both humans and animals. Based on antigenic differences observed between the nucleoprotein (NP) and matrix protein (M), influenza virus can be categorized into four distinct types—influenza A virus (IAV), influenza B virus (IBV), influenza C virus (ICV), and influenza D virus (IDV). Among these, IAV is particularly significant due to its role in severe epidemics affecting both human populations and domestic animals, serving as a primary causative agent of acute infectious respiratory disease [1]. The natural reservoirs for the virus are primarily waterfowl; however, evolutionary processes have expanded its host range to include various avian species, as well as several mammals such as swine, horses, and humans [2]. Historically, pandemic outbreaks have resulted in considerable economic losses within the livestock and poultry industries while simultaneously posing substantial threats to public health and safety.

The antiviral innate immune response of the host serves as a critical first line of defense, rapidly detecting and countering viral invasions. This process commences with the recognition of pathogen-associated molecular patterns (PAMPs) by pattern recognition receptors (PRRs), including Toll-like receptors, RIG-I-like receptors (RLRs), and NOD-like receptors (NLRs). Among these, RLRs such as RIG-I and MDA5 play pivotal roles; upon binding to viral RNA, they interact with mitochondrial antiviral signaling protein (MAVS) [3]. This interaction triggers a signaling cascade that activates interferon regulatory factors (IRFs), particularly IRF3 and IRF7. Once activated, these IRFs translocate to the nucleus and initiate the expression of interferons (IFNs), which are essential for establishing an antiviral state in both infected cells and their neighboring counterparts [4,5]. Type I and type III interferons signal through their respective receptor complexes, thereby activating the JAK/STAT pathway. In this context, Janus kinases (JAKs) phosphorylate signal transducer and activator of transcription (STAT) proteins, leading to dimerization, before entering the nucleus to induce the expression of interferon-stimulated genes (ISGs) [6,7,8]. These ISGs encode proteins that execute various antiviral functions including the inhibition of viral replication, the promotion of apoptosis, and the enhancement of antigen presentation to facilitate adaptive immune responses [9,10].

Long non-coding RNAs (lncRNAs) are a class of non-protein-coding transcripts exceeding 200 nucleotides in length. They play pivotal roles in the regulation of gene expression and a variety of cellular processes, including chromatin remodeling, transcriptional regulation, and post-transcriptional modifications [11,12]. LncRNAs have been found to act as molecular scaffolds, guides for chromatin modifiers, and regulators of mRNA stability and translation [13,14].

In the realm of viral infections, including influenza, lncRNAs have emerged as key regulators of host–virus interactions. They can modulate the host’s immune response, influence viral replication, and affect the pathogenesis of viral diseases. For instance, during influenza virus infection, lncRNA-155 serves a dual function by acting as a precursor for miR-155 while simultaneously modulating the innate antiviral immune response through the intricate regulation of IRF3 activation [15]. LncRNA GAPLINC is positively regulated by ATG7 and enhances influenza virus replication by inhibiting IRF3 activation, which subsequently leads to reduced interferon production and the suppression of the host’s antiviral immune response [16]. Conversely, lncRNA LINC02574 is induced by interferons and establishes a positive feedback loop within the innate antiviral immune system of the host, thereby inhibiting influenza virus replication [17].

In this study, we observed a significant downregulation of lncRNA THRIL expression following influenza virus infection. Moreover, our findings indicate that THRIL promotes influenza virus replication by inhibiting the antiviral innate immune response. The luciferase reporter assay revealed that THRIL may modulate host innate immunity through the suppression of IRF3 activation. This research lays a scientific foundation for elucidating the mechanisms by which the influenza virus evades host natural immune defenses and clarifies its pathogenic processes. These results offer novel insights and theoretical groundwork for developing more effective strategies to prevent and control influenza virus infections.

## 2. Materials and Methods

### 2.1. Cells, Reagents, and Plasmids

A549, MDCK, and 293T cells were purchased from American Type Culture Collection (Manassas, VA, USA). The cells were cultured in Dulbecco’s modified Eagle’s medium (DMEM) containing 10% fetal bovine serum (FBS), supplemented with 100 U/mL penicillin–streptomycin (Beyotime Biotechnology, Shanghai, China) at 37 °C in a humidified atmosphere of 5% CO_2_. IFN-β and IL-29 was purchased from Peprotech (London, UK), and poly (I:C) was obtained from Invivogen (San Diego, CA, USA). LPS was purchased from Sigma Aldrich (St. Louis, MO, USA). Anti-IRF3 and anti-p-IRF3 were purchased from Cell Signaling Technology (Danvers, MA, USA), and anti-β-actin was purchased from TransGen Biotech (Beijing, China). Luciferase reporter plasmids including IFN-β promoter reporter plasmid (IFN-β-Luc) and pRL-TK were gifts from Dr. Chunfu Zheng (Fujian Medical University, Fuzhou, China). Other plasmids expressing RIG-I, MAVS, TBK1, and IRF3 were constructed as described previously [18].

### 2.2. Viruses and Viral Infection

The influenza virus strains utilized in this study include A/WSN/33 (H1N1) (WSN), A/Puerto Rico/8/1934 (H1N1) (PR8), A/California/04/2009 (H1N1) (CA04), and A/Chicken/Fujian/MQ01/2015 (H9N2). Influenza viruses and Sendai virus (SeV) were passaged in specific-pathogen-free (SPF) chicken embryos, and Pseudorabies virus (PRV) was propagated in PK15 cells, as previously described [19]. For infections with influenza viruses and SeV, cells were cultured to confluence in six-well plates and were subsequently infected with the virus at the indicated multiplicity of infection (MOI). Following a one-hour adsorption period, the cells were washed with phosphate-buffered saline (PBS) and maintained in serum-free DMEM supplemented with 2 μg/mL of trypsin. For PRV infection, the virus was added to cells in serum-free medium at the indicated MOI for one hour, followed by PBS washing and culture in DMEM supplemented with 2% FBS.

### 2.3. RT-PCR and Quantitative Real-Time PCR (qRT-PCR)

Total RNA was extracted from cells using Trizol reagent (TIANGEN, Beijing, China). cDNA synthesis was performed with the HiScript III 1st Strand cDNA Synthesis Kit (Vazyme, Nanjing, China). Subsequently, PCR and qPCR were conducted using Taq DNA polymerase (GenStar, Beijing, China) and SYBR Green Master Mix (Vazyme, Nanjing, China), respectively. GAPDH served as a reference gene for internal standardization. The qRT-PCR data are presented as normalized ratios calculated automatically using the ΔΔCT method via the LightCycler system (Roche, Switzerland).

### 2.4. Western Blotting

Cell lysates were prepared and Western blotting was conducted as previously described [20]. In brief, harvested cells were lysed using RIPA buffer (Cell Signaling Technology, Danvers, MA, USA) supplemented with protease inhibitors. The samples were subjected to sodium dodecyl sulfate-polyacrylamide gel electrophoresis (SDS-PAGE) and were subsequently transferred onto a nitrocellulose membrane. Following this, the membrane was blocked and probed with the specified antibodies. Protein detection was performed using enhanced chemiluminescence (ECL) procedures in accordance with the manufacturer’s instructions.

### 2.5. Plaque Assay and Hemagglutinin Assay

For the plaque assay, MDCK cells were exposed to serially diluted supernatants from virus-infected cell cultures at 37 °C for 1 h. Following this, the cells underwent a rinse with PBS and were covered with α-minimal essential medium containing 1.5% low-melting-point agarose and 2 μg/mL TPCK-treated trypsin. After a subsequent incubation period of 72 h at 37 °C, the cells were fixed with 4% formaldehyde, and plaques were stained and quantified. For hemagglutinin assay, the supernatants were serially diluted and mixed with an equal volume of 0.5% chicken erythrocytes. Viral titers were determined after a 30 min incubation at room temperature.

### 2.6. Generation of Cell Lines

THRIL-knockdown and control cell lines were established by infecting A549 cells with lentiviruses that express specific shRNAs targeting THRIL or luciferase, utilizing the pSIH-H1-GFP vector as previously described [21]. The sequence employed for the shRNA targeting of THRIL was ACCTCACCCACCAATCCCTAA. Additionally, cell lines overexpressing THRIL or an empty vector were generated through the infection of A549 cells with lentiviruses encoding these genes in the pNL-GFP vector, following previously outlined methods [21].

### 2.7. Dual Luciferase Reporter Assay

293T cells were seeded in 24-well culture plates and were transfected with the IFN-β-Luc reporter plasmid, pRL-TK, pNL-THRIL, or empty vector (EV), together with the plasmid encoding RIG-I, MAVS, TBK1, or IRF3. After 24 h of transfection, the cells were lysed, followed by the dual-luciferase activity assays using the dual-luciferase reporter assay kit (Promega, WI, USA) according to the manufacturer’s instructions.

### 2.8. Statistical Analysis

Statistical analysis was performed using Student’s *t* test. Data represent the mean values ± SD from three independent experiments. A level of *p* < 0.05 was considered statistically significant.

## 3. Results

### 3.1. Infection of Multiple Viruses Downregulates the Expression of THRIL

**Figure 1:**
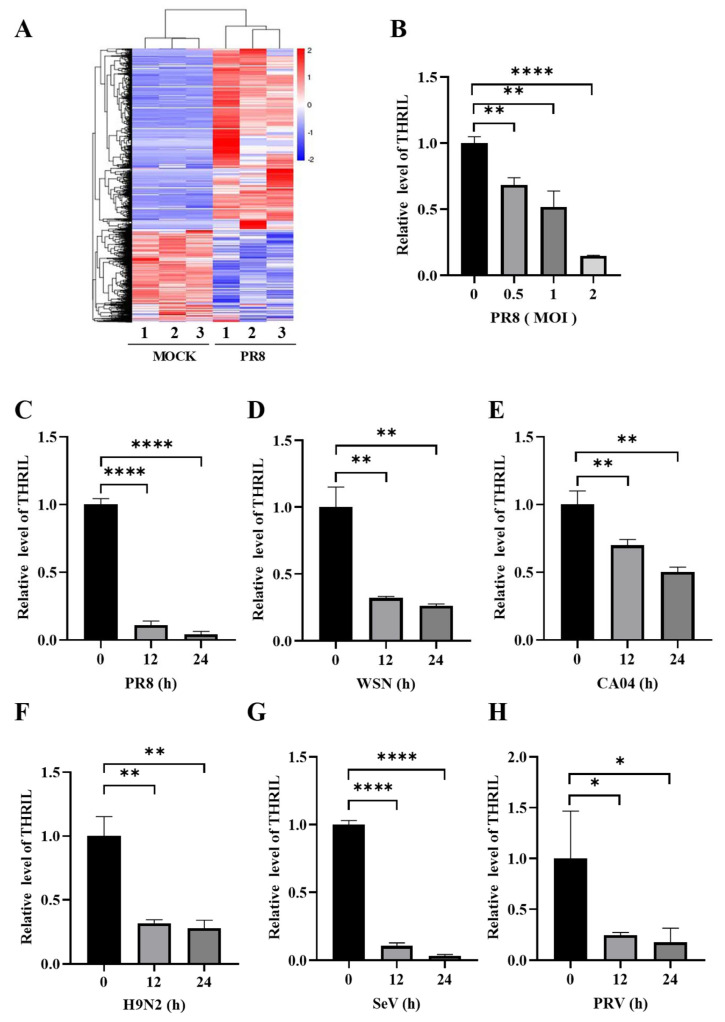
The infection of multiple viruses downregulates the expression of THRIL. (**A**) RNA-seq analysis of A549 cells infected with the PR8 influenza virus for 12 h. The figure presents a heat map derived from the RNA-seq results. (**B**) A549 cells were infected with different multiplicities of infection of the PR8 virus for 12 h. The expression level of THRIL was detected using qRT-PCR. (**C**–**H**) A549 cells were infected with PR8 (**C**), WSN (**D**), CA04 (**E**), H9N2 (**F**), SeV (**G**), and PRV (**H**) for the indicated time period; then, the expression of THRIL was examined using qRT-PCR. Data are represented as mean ± SD; *n* = 3; * *p* < 0.05, ** *p* < 0.01, **** *p* < 0.0001.

To elucidate the functional lncRNAs implicated in influenza virus infection, we performed RNA sequencing analysis (GSE252713) on A549 cells following infection with the PR8 strain of the influenza virus. Our findings revealed that hundreds of thousands of lncRNAs were dysregulated post-infection (Figure 1A). Among these, we identified lncRNA THRIL as a notable candidate. Subsequently, we infected A549 cells at various multiplicities of infection (MOIs) with the PR8 virus and assessed THRIL expression patterns via qRT-PCR at 12 h post-infection. Remarkably, lncRNA THRIL exhibited a significant reduction in expression after viral exposure (Figure 1B). Furthermore, we evaluated the impact of diverse influenza strains and other viruses—including H1N1 subtypes (PR8, WSN, and CA04), H9N2 subtypes, Sendai virus (SeV), and pseudorabies virus (PRV)—on THRIL expression levels in A549 cells at time points of 0, 12, and 24 h. Validation through qRT-PCR confirmed a substantial decrease in THRIL expression correlating with extended infection durations (Figure 1C–H), suggesting a consistent downregulatory effect across different viral strains. Collectively, these results indicate that THRIL expression is negatively regulated in response to both RNA and DNA viral infections within A549 cells, underscoring its potential role in host antiviral defense mechanisms.

### 3.2. Interferon Treatment Decreased the Expression of THRIL

**Figure 2:**
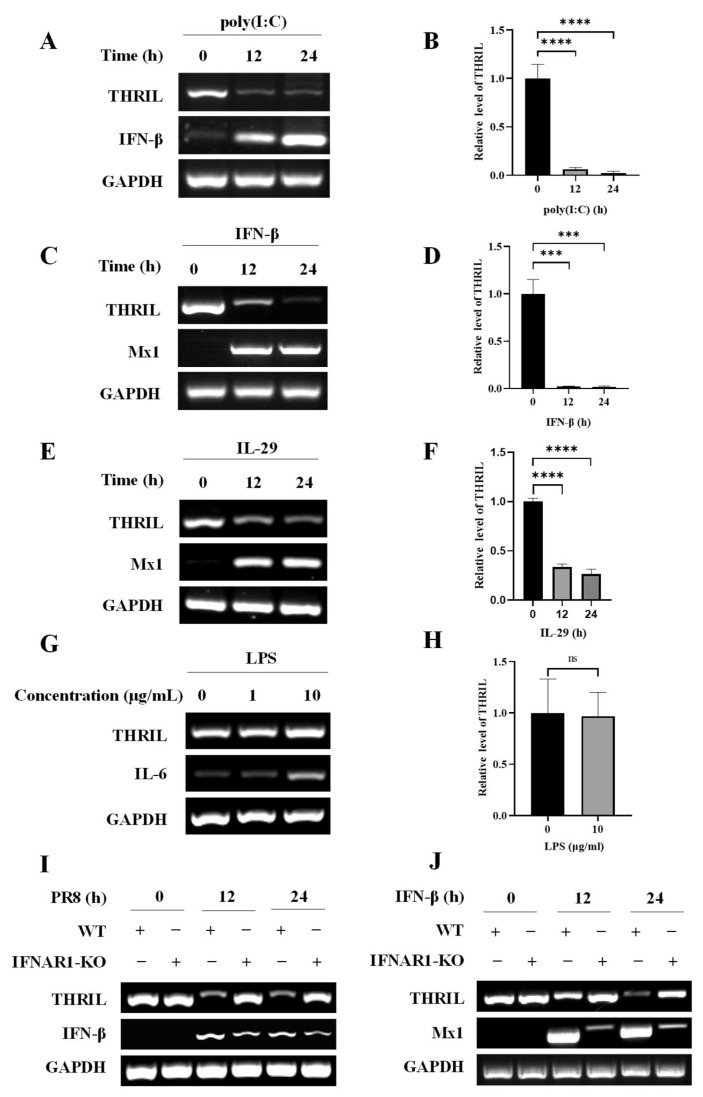
Interferon treatment decreased the expression of THRIL. (**A**,**B**) A549 cells were stimulated with poly (I:C) for the indicated time period; then, THRIL expression was detected using RT-PCR (**A**) and qRT-PCR (**B**). (**C**–**F**) A549 cells were stimulated with IFN-β (**C**,**D**) and IL-29 (**E**,**F**) for the indicated time period; then, THRIL expression was examined using RT-PCR (**C**,**E**) and qRT-PCR (**D**,**F**). (**G**,**H**) A549 cells were treated with LPS at the indicated concentrations for 6 h. The expression of THRIL was detected using RT-PCR (**G**) and qRT-PCR (**H**). (**I**,**J**) IFNAR1 knockout (KO) and wild-type (WT) A549 cells were infected with the PR8 virus (**I**) or treated with IFN-β (**J**) for the indicated time period; then, THRIL expression was examined using RT-PCR. Data are represented as mean ± SD; *n* = 3; *** *p* < 0.001, **** *p* < 0.0001. “ns” represents no significance.

To investigate the regulation of THRIL expression in response to influenza virus infection, we employed poly (I:C), a synthetic analog of viral double-stranded RNA, to stimulate A549 cells. The expression levels of THRIL were assessed using RT-PCR and qRT-PCR techniques. Our findings revealed a consistent decrease in THRIL expression following poly (I:C) treatment (Figure 2A,B). It is well established that poly (I:C) serves as a potent inducer of interferons; thus, we further examined whether the downregulation of THRIL during viral infection correlated with elevated levels of type I and type III interferons. As anticipated, stimulation with either IFN-β or IL-29 resulted in a significant reduction in THRIL expression within A549 cells (Figure 2C–F). Additionally, we evaluated the influence of inflammatory factors on THRIL expression and found that treatment with LPS did not yield any significant effects on its expression levels (Figure 2G,H). Interferons exert their antiviral effects by binding to the receptors located on cell membrane surfaces, thereby activating the transcriptional cascade for various antiviral ISGs. In this study, we utilized IFNAR1 knockout (IFNAR1 KO) A549 cells and exposed them to either influenza virus infection or IFN-β stimulation to conduct subsequent analyses. Notably, the downregulation of THRIL induced by either influenza virus or IFN-β was abolished in IFNAR1 KO cells compared to their control counterparts (Figure 2I,J). These findings suggest that THRIL expression is modulated by interferon signaling following viral infection.

### 3.3. THRIL Promotes the Replication of Influenza Virus

**Figure 3:**
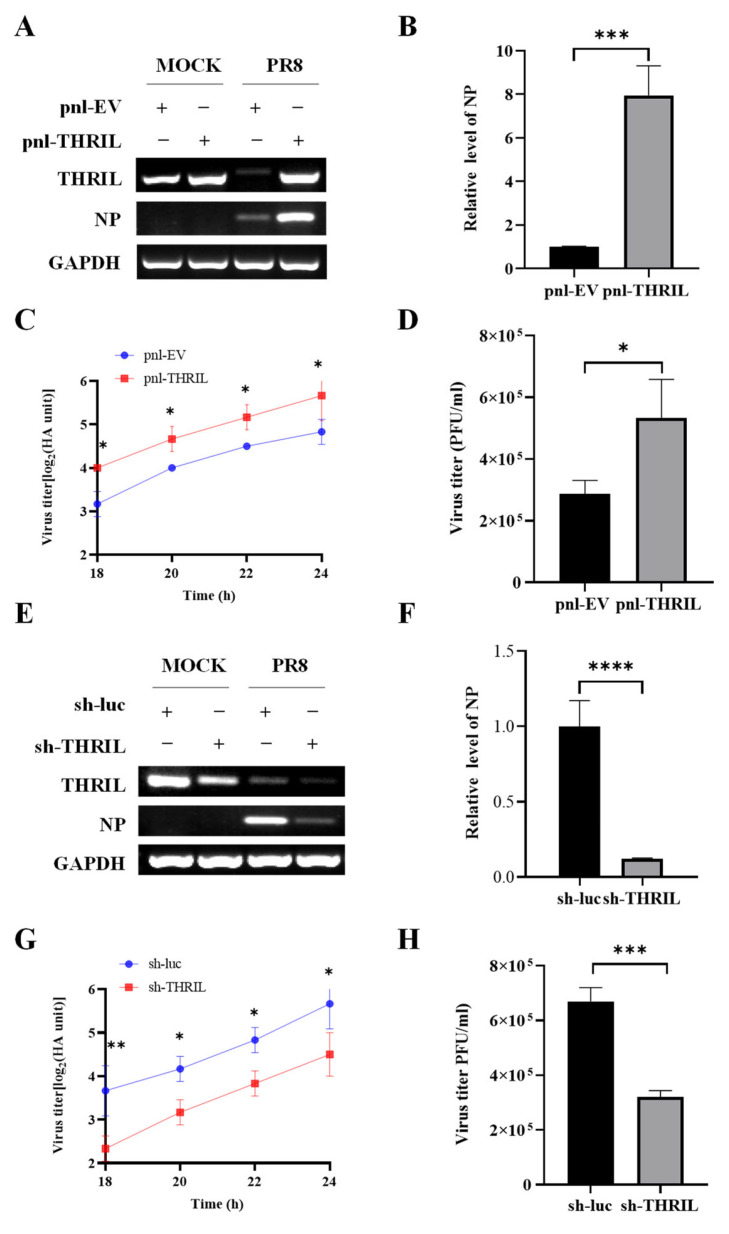
THRIL promotes the replication of influenza virus. (**A**,**B**) A549 cells stably expressing THRIL or empty vector (EV) were infected with or without PR8 virus (MOI = 1) for 24 h. After infection, the mRNA levels of viral NP were assessed using RT-PCR (**A**) and qRT-PCR (**B**). (**C**) The replication kinetics of the PR8 virus (MOI = 1) in THRIL-overexpressing A549 cells and control cells were detected using the hemagglutinin (HA) assay. (**D**) THRIL-overexpressing A549 cells and control cells were infected with the PR8 virus (MOI = 1) for 24 h. Viral titers in cell culture supernatants were examined using the plaque assay. (**E**,**F**) A549 cells stably expressing shRNA targeting THRIL (sh-THRIL) or luciferase control (sh-luc) were infected with the PR8 virus (MOI = 1) for 24 h. After infection, the mRNA levels of viral NP were assessed using RT-PCR (**E**) and qRT-PCR (**F**). (**G**) The replication kinetics of the PR8 virus (MOI = 1) in THRIL-knockdown A549 cells and control cells were detected using an HA assay. (**H**) THRIL-knockdown A549 cells and control cells were infected with the PR8 virus (MOI = 1) for 24 h. Viral titers in cell culture supernatants were examined using the plaque assay. Data are represented as mean ± SD; *n* = 3; * *p* < 0.05, ** *p* < 0.01, *** *p* < 0.001, **** *p* < 0.0001.

In our subsequent analysis, we identified that THRIL significantly influences the replication of the influenza virus in A549 cells. Specifically, when THRIL was ectopically overexpressed in these cells, there was a pronounced upregulation in the expression levels of the viral nucleoprotein (NP) (Figure 3A,B). This increase in NP expression correlated with a substantial increase in viral load, as demonstrated by the elevated viral titers observed in the cell culture supernatant (Figure 3C,D). To further elucidate the role of THRIL in facilitating influenza virus replication, we performed experiments involving the specific silencing of THRIL expression in A549 cells. Under these conditions, we noted a significant reduction in NP levels (Figure 3E,F). Correspondingly, the viral load present in the supernatant was also markedly diminished (Figure 3G,H), suggesting that THRIL is a positive regulator of influenza virus replication.

### 3.4. THRIL Negatively Regulates the IAV-Induced Expression of Interferons

**Figure 4:**
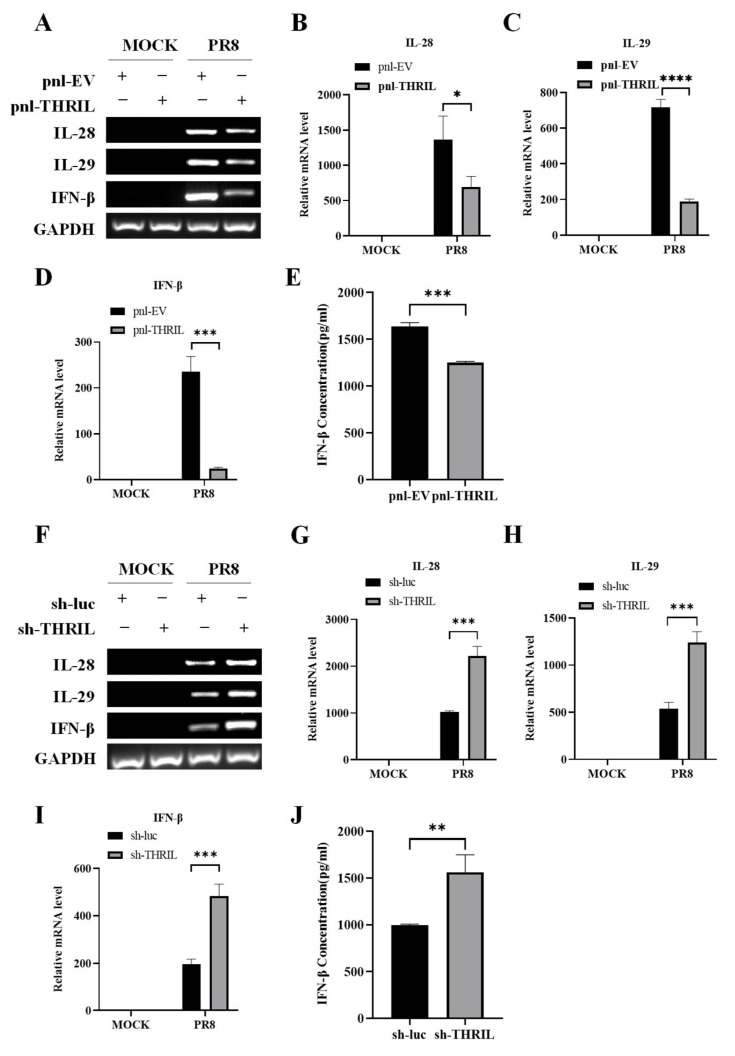
THRIL negatively regulates the IAV-induced expression of interferons. (**A**–**D**) THRIL-overexpressing A549 cells and control cells were infected with the PR8 virus (MOI = 1) for 24 h. After infection, the mRNA levels of IL-28, IL-29, and IFN-β were determined using RT-PCR (**A**) and qRT-PCR (**B**–**D**). (**E**) THRIL-overexpressing A549 cells and control cells were treated as described in (**A**–**D**). IFN-β levels in the cell culture supernatants were measured using ELISA. (**F**–**I**) THRIL-knockdown A549 cells and control cells were infected with the PR8 virus (MOI = 1) for 24 h. After infection, the mRNA levels of IL-28, IL-29, and IFN-β were determined using RT-PCR (**F**) and qRT-PCR (**G**–**I**). (**J**) THRIL-knockdown A549 cells and control cells were treated as described in (**F**–**I**). IFN-β levels in the cell culture supernatants were measured using ELISA. Data are represented as mean ± SD; *n* = 3; * *p* < 0.05, ** *p* < 0.01, *** *p* < 0.001, **** *p* < 0.0001.

Given that THRIL has been shown to enhance the replication of influenza virus, it is essential to investigate its effects on the host’s innate immune response. The innate immune system acts as the primary line of defense against viral infections, and elucidating THRIL’s role in modulating this response is critical for understanding the dynamics of virus–host interactions. We conducted a series of experiments utilizing the pnl-THRIL cell line, which stably overexpresses THRIL, and exposed it to the PR8 influenza virus. Subsequent analyses demonstrated a significant reduction in the expression levels of key interferons, including type I interferon IFN-β and type III interferons IL-28 and IL-29, compared to the control cells (Figure 4A–D). In line with the changes in IFN mRNA expression, there was an observed decrease in IFN-β production in the culture supernatants (Figure 4E). These findings were corroborated by the contrasting results from the sh-THRIL cell line, where the specific knockdown of THRIL expression led to a marked upregulation of interferon mRNA expression and IFN-β secretion upon PR8 influenza virus infection (Figure 4F–J). Collectively, these findings suggest that THRIL exerts a suppressive effect on interferon regulation following viral infection.

### 3.5. THRIL Exerts an Inhibitory Effect on the Expression of Multiple Essential Antiviral ISGs

**Figure 5:**
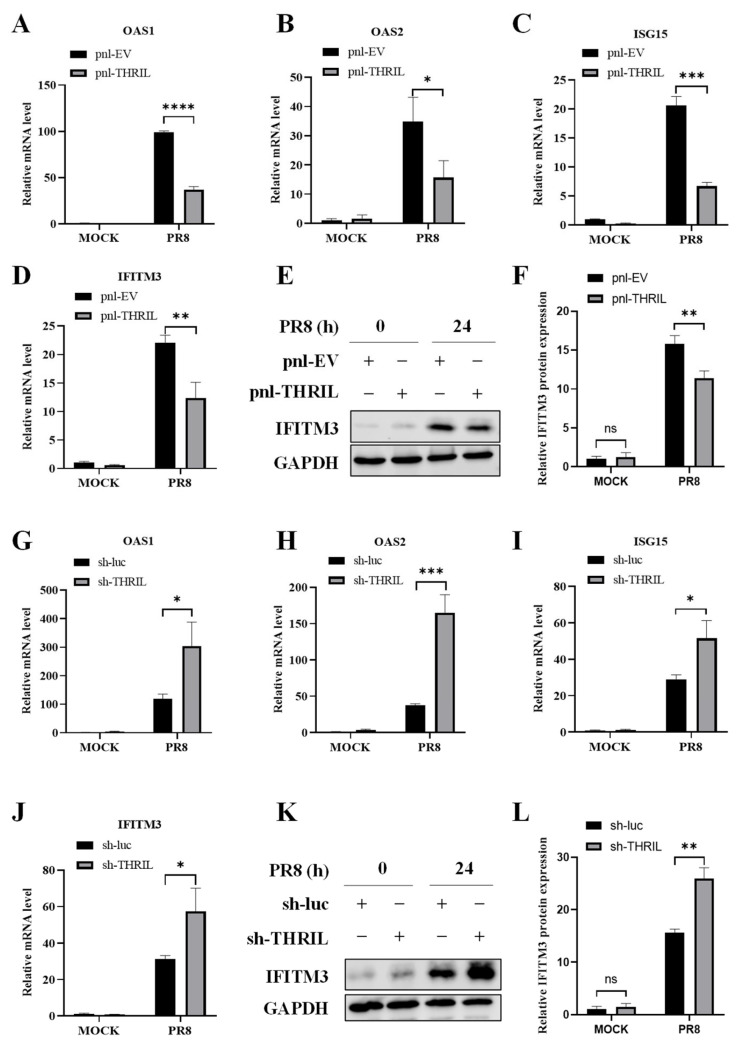
THRIL inhibits the expression of several antiviral ISGs. (**A**–**D**) THRIL-overexpressing A549 cells and control cells were infected with the PR8 virus (MOI = 1) for 24 h. After infection, the mRNA levels of OAS1 (**A**), OAS2 (**B**), ISG15 (**C**), and IFITM3 (**D**) were determined using qRT-PCR. (**E**,**F**) THRIL-overexpressing A549 cells and control cells were treated as described in (**A**–**D**). The protein expression of IFITM3 in cells was detected using Western blotting (**E**). The relative levels of IFITM3 in (**E**) were quantitated using densitometry and were normalized to GAPDH levels (**F**). (**G**–**J**) THRIL-knockdown A549 cells and control cells were infected with the PR8 virus (MOI = 1) for 24 h. After infection, the mRNA levels of OAS1 (**G**), OAS2 (**H**), ISG15 (**I**), and IFITM3 (**J**) were determined using qRT-PCR. (**K**,**L**) THRIL-knockdown A549 cells and control cells were treated as described in (**G**–**J**). The protein expression of IFITM3 in cells was detected using Western blotting (**K**). The relative levels of IFITM3 in (**K**) were quantitated using densitometry and were normalized to GAPDH levels (**L**). Data are represented as mean ± SD; *n* = 3; * *p* < 0.05, ** *p* < 0.01, *** *p* < 0.001, **** *p* < 0.0001. “ns” represents no significance.

ISGs are a set of genes induced by interferons and are crucial for the host’s defense mechanisms against viral infections. In our subsequent investigation, we extended our focus to the expression of key ISGs that are crucial in the antiviral defense cascade. Specifically, we scrutinized the expression profiles of OAS1, OAS2, ISG15, and IFITM3, which are well characterized for their roles in restricting viral replication and modulating immune signaling pathways. Our results revealed that in cells with elevated THRIL expression, the mRNA expression levels of these ISGs were significantly diminished (Figure 5A–D). Additionally, we assessed the protein level of IFITM3 and observed a marked decrease in IFITM3 protein expression in THRIL-overexpressing cells (Figure 5E,F). Conversely, in cells with THRIL knockdown, both the mRNA expression of these ISGs and the protein level of IFITM3 were notably upregulated (Figure 5G–L). Taken together, these results suggest that THRIL suppresses host innate immunity, likely through negatively regulating the expression of IFNs and thereby reducing the levels of some critical ISGs.

### 3.6. THRIL Attenuates Host Innate Immunity by Inhibiting IRF3 Activity

**Figure 6:**
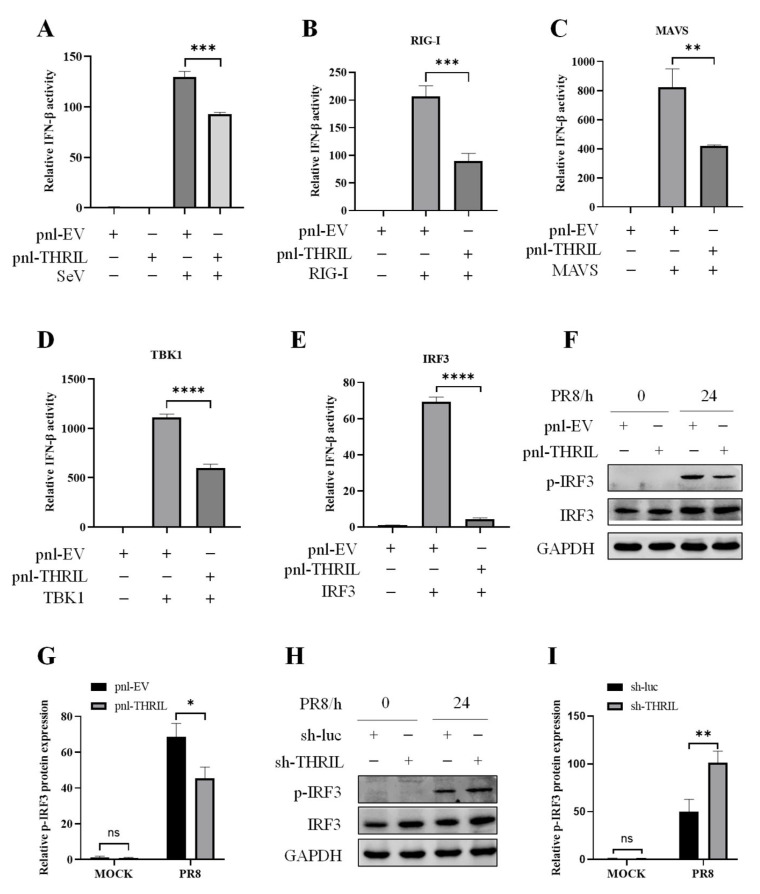
THRIL inhibits host innate immunity through targeting IRF3. (**A**) 293T cells were co-transfected with 500 ng IFN-β-Luc, 50 ng pRL-TK, and 500 ng empty vector (EV) or THRIL-expressing plasmids for 24 h. After that, cells were infected with SeV, and dual-luciferase activity was examined at 12 h post-infection. (**B**–**E**) 293T cells were co-transfected with 500 ng IFN-β-Luc, 50 ng pRL-TK, and 500 ng THRIL-expressing plasmid or EV, along with 300 ng RIG-I (**B**), MAVS (**C**), TBK1 (**D**), or IRF3 (**E**). Luciferase activity was detected 24 h post-transfection. (**F**–**I**) THRIL-overexpressing (**F**,**G**) or THRIL-knockdown (**H**,**I**) A549 cells and control cells were infected with the PR8 virus (MOI = 1) for 24 h. After infection, the phosphorylation levels of IRF3 were examined using Western blotting (**F**,**H**). The relative levels of p-IRF3 in (**F**,**H**) were quantitated using densitometry and were normalized to GAPDH levels (**G**,**I**). Data are represented as mean ± SD; n = 3; * *p* < 0.05, ** *p* < 0.01, *** *p* < 0.001, **** *p* < 0.0001. “ns” represents no significance.

In order to elucidate the mechanism through which THRIL suppresses the expression of IFN-β, we investigated the specific point in the signaling cascade where THRIL exerts its inhibitory action. To achieve this, the THRIL-expressing plasmid and empty vector plasmid were co-transfected into 293T cells with IFN-β-luc and pRL-TK, respectively, for 24 h; then, these cells were infected with SeV. Cell samples were collected 12 h later for the dual-luciferase reporter assay. The results showed that THRIL inhibited the activity of the IFN-β promoter during SeV infection (Figure 6A). Subsequently, 293T cells were co-transfected with IFN-β-luc and pRL-TK, along with expression plasmids for RIG-I, MAVS, TBK1, or IRF3, in the presence of either the THRIL-expressing vector or an empty vector. The subsequent luciferase reporter assay revealed that THRIL significantly attenuated the activation of the IFN-β promoter through each of these signaling components (Figure 6B–E), indicating that THRIL may impede the IFN-β signaling pathway at the stage of IRF3 activation. To further validate these findings, we assessed IRF3 activity in cells with either THRIL overexpression or knockdown. The results demonstrated a marked reduction in IRF3 phosphorylation levels in THRIL-overexpressing cells (Figure 6F,G), whereas an increase in IRF3 phosphorylation was observed in cells with THRIL knockdown (Figure 6H,I). Collectively, these data suggest that THRIL inhibits interferon production by blocking IRF3 activation, thereby playing a negative regulatory role in the host’s innate immune response.

## 4. Discussion

LncRNAs have emerged as critical regulators in various biological processes, including immune responses and viral infections [22,23]. THRIL, the TNF-α and hnRNPL related immunoregulatory lincRNA, has been previously reported to play a crucial role in regulating the inflammatory response. It is transcriptionally activated by AP-1 and stabilized by METTL14-mediated m6A modification, which accelerates LPS-evoked acute injury in alveolar epithelial cells [24]. In addition, the expression level of THRIL was significantly upregulated in moderate and severe COVID-19 patients compared to the control group, with higher levels observed in severe patients [25]. This indicates that THRIL could be considered as a potential circulating biomarker of SARS-CoV-2 infection and disease severity. In this study, we identified that THRIL is downregulated upon influenza virus infection, suggesting a potential regulatory function in the life cycle of the influenza virus.

Interferons are a critical component of the innate immune response and play a significant role in the host’s defense against viral infections. LncRNAs have emerged as important regulators within this response, and there is growing evidence that they can be influenced by interferon signaling and, in turn, modulate the interferon response itself [26]. For instance, lncRNA-ISIR was identified as an ISG and was transcribed via STAT1 in response to type I interferon stimulation. The induced lncRNA-ISIR interacted with and activated IRF3 by disrupting the inhibitory influence of Fli-1, thereby enhancing the production of interferons [27]. Additionally, lncRNA PCBP1-AS1 could be upregulated following treatment with either type I or type III interferons; this increase in PCBP1-AS1 promoted influenza virus replication through the encoding of a small protein known as PESP [19]. Both lncRNAs ISR and LINC02574 were also induced by interferon treatment, triggering the production of interferons and positively regulating the innate immune response to inhibit influenza A virus replication [17,28]. Our current study further elucidated the regulatory role of lncRNAs in response to influenza virus infection and interferon treatment. We observed a significant downregulation in THRIL expression following stimulation with poly (I:C), as well as IFN-β and IL-29, suggesting that the transcriptional regulation or RNA stabilization of THRIL may be impacted. Moreover, the lack of significant effects on THRIL expression following treatment with IL-6 and LPS indicates that the regulation of THRIL is likely specific to the interferon response rather than a general inflammatory response. However, the underlying mechanisms through which interferons regulate lncRNA expression remain poorly understood and require further investigation.

Several lncRNAs have been recognized as integral components of the host’s immune response to viral infections, particularly influenza. They can modulate the interferon pathway at multiple levels, from the activation of transcription factors to the regulation of antiviral gene expression [29,30]. THRIL, whose expression was inhibited by interferon, also played a significant role in modulating the host’s innate immune response to influenza virus infection. By utilizing a stable cell line overexpressing THRIL and exposing it to influenza virus, we observed a significant reduction in the expression of type I and type III interferons, as well as some key ISGs. The contrasting results from the THRIL-knockdown cell line further supported the suppressive role of THRIL in interferon and ISG regulation. These findings suggest that THRIL contributes to the suppression of the antiviral state, thereby facilitating influenza virus replication. Understanding the precise mechanisms by which THRIL exerts its effects on interferon regulation could provide valuable insights into the pathogenesis of influenza and potentially uncover new targets for therapeutic intervention.

LncRNAs play a crucial role in modulating interferon production by interacting with various components of the interferon signaling pathway. For example, Lnczc3h7a acts as a molecular scaffold that enhances the antiviral innate immune response through the stabilization of the RIG-I-TRIM25 complex, thereby facilitating TRIM25-mediated K63-linked ubiquitination of RIG-I and promoting downstream interferon synthesis [31]. LncRNA-GM interacts with glutathione S-transferase M1 (GSTM1), inhibiting its interaction with TBK1 kinase, which is essential for activating IRF3 and subsequent IFN-I production [32]. Additionally, lncRNA Malat1 binds to TDP43 within the nucleus, preventing the caspase-3-mediated cleavage of TDP43 into TDP35. The resultant TDP35 enhances nuclear IRF3 levels by degrading Rbck1 pre-mRNA, thus averting the proteasomal degradation of IRF3 during viral infection and selectively promoting antiviral IFN-I production [33]. In our study, we demonstrated that THRIL attenuates IRF3 activation, consequently inhibiting interferon production; however, further detailed data are required to elucidate the precise molecular mechanisms underlying THRIL’s inhibition of IRF3 activation. It may exert indirect regulation on IRF3 activity via targeting other molecules or may directly target IRF3 to inhibit its activation. Furthermore, the regulatory role of THRIL in host innate immunity suggests its potential involvement in the replication of various viruses.

**Figure 7:**
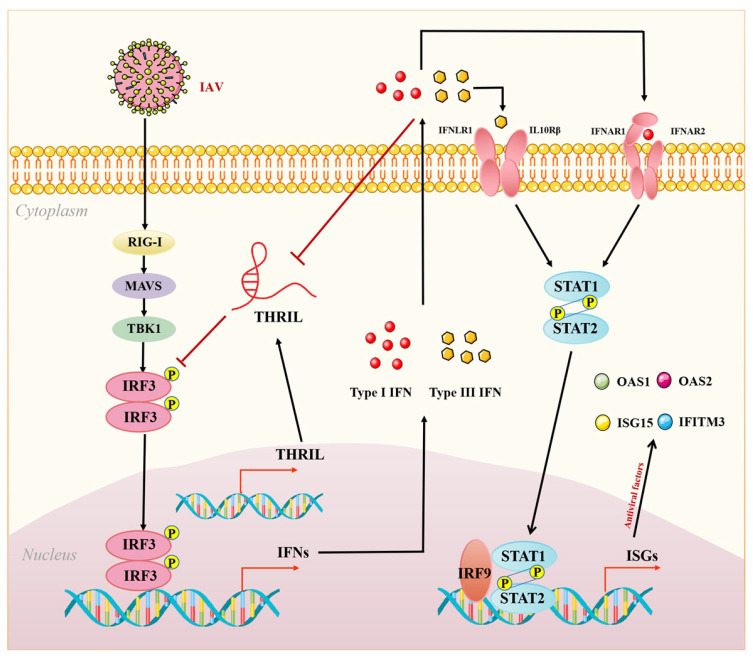
A schematic representation of THRIL inhibiting the host innate immune response by blocking IRF3 activation.

In conclusion, our study reveals a novel aspect of THRIL regulation within the context of influenza virus infection and its feedback modulation of interferon expression (Figure 7). A marked downregulation in THRIL expression following IAV infection indicates its potential as a biomarker for the early detection and evaluation of disease severity. Moreover, the role of THRIL in promoting viral replication and suppressing host immune responses underscores its potential as a therapeutic target. By modulating THRIL expression, we may enhance the host’s innate immune response, thereby offering a promising strategy for treating IAV infections. Further research is necessary to elucidate the mechanistic details of the inter-regulation between THRIL and the host immune system, providing an important scientific basis for the clinical application of THRIL.

## Data Availability

All relevant data are within the manuscript and figures. The data from RNA-seq have been deposited in the GEO public database under the accession number GSE252713.

## References

[1] Flerlage T., Boyd D.F., Meliopoulos V., Thomas P.G., Schultz-Cherry S. (2021). Influenza virus and SARS-CoV-2: Pathogenesis and host responses in the respiratory tract. Nat. Rev. Microbiol..

[2] Wille M., Holmes E.C. (2020). The Ecology and Evolution of Influenza Viruses. Cold Spring Harb. Perspect. Med..

[3] Kawai T., Akira S. (2006). Innate immune recognition of viral infection. Nat. Immunol..

[4] Kagan J.C. (2012). Signaling organelles of the innate immune system. Cell.

[5] Iwasaki A., Pillai P.S. (2014). Innate immunity to influenza virus infection. Nat. Rev. Immunol..

[6] Lazear H.M., Schoggins J.W., Diamond M.S. (2019). Shared and Distinct Functions of Type I and Type III Interferons. Immunity.

[7] Villarino A.V., Kanno Y., O’Shea J.J. (2017). Mechanisms and consequences of Jak-STAT signaling in the immune system. Nat. Immunol..

[8] Gonzalez-Navajas J.M., Lee J., David M., Raz E. (2012). Immunomodulatory functions of type I interferons. Nat. Rev. Immunol..

[9] Duggal N.K., Emerman M. (2012). Evolutionary conflicts between viruses and restriction factors shape immunity. Nat. Rev. Immunol..

[10] Sadler A.J., Williams B.R. (2008). Interferon-inducible antiviral effectors. Nat. Rev. Immunol..

[11] Mattick J.S., Amaral P.P., Carninci P., Carpenter S., Chang H.Y., Chen L.L., Chen R., Dean C., Dinger M.E., Fitzgerald K.A. (2023). Long non-coding RNAs: Definitions, functions, challenges and recommendations. Nat. Rev. Mol. Cell Biol..

[12] Ferrer J., Dimitrova N. (2024). Transcription regulation by long non-coding RNAs: Mechanisms and disease relevance. Nat. Rev. Mol. Cell Biol..

[13] Chen L.L., Kim V.N. (2024). Small and long non-coding RNAs: Past, present, and future. Cell.

[14] Yao R.W., Wang Y., Chen L.L. (2019). Cellular functions of long noncoding RNAs. Nat. Cell Biol..

[15] Rai K.R., Liao Y., Cai M., Qiu H., Wen F., Peng M., Wang S., Liu S., Guo G., Chi X. (2022). MIR155HG Plays a Bivalent Role in Regulating Innate Antiviral Immunity by Encoding Long Noncoding RNA-155 and microRNA-155-5p. mBio.

[16] Chen B., Guo G., Wang G., Zhu Q., Wang L., Shi W., Wang S., Chen Y., Chi X., Wen F. (2024). ATG7/GAPLINC/IRF3 axis plays a critical role in regulating pathogenesis of influenza A virus. PLoS Pathog..

[17] Zhang Y., Chi X., Hu J., Wang S., Zhao S., Mao Y., Peng B., Chen J., Wang S. (2023). LncRNA LINC02574 Inhibits Influenza A Virus Replication by Positively Regulating the Innate Immune Response. Int. J. Mol. Sci..

[18] Hong J., Chi X., Yuan X., Wen F., Rai K.R., Wu L., Song Z., Wang S., Guo G., Chen J.L. (2022). I226R Protein of African Swine Fever Virus Is a Suppressor of Innate Antiviral Responses. Viruses.

[19] Chi X., Huang G., Wang L., Zhang X., Liu J., Yin Z., Guo G., Chen Y., Wang S., Chen J.L. (2024). A small protein encoded by PCBP1-AS1 is identified as a key regulator of influenza virus replication via enhancing autophagy. PLoS Pathog..

[20] Wang S., Jiang N., Shi W., Yin H., Chi X., Xie Y., Hu J., Zhang Y., Li H., Chen J.L. (2021). Co-infection of H9N2 Influenza A Virus and Escherichia coli in a BALB/c Mouse Model Aggravates Lung Injury by Synergistic Effects. Front. Microbiol..

[21] Wang S., Chi X., Wei H., Chen Y., Chen Z., Huang S., Chen J.L. (2014). Influenza A virus-induced degradation of eukaryotic translation initiation factor 4B contributes to viral replication by suppressing IFITM3 protein expression. J. Virol..

[22] Vierbuchen T., Fitzgerald K.A. (2021). Long non-coding RNAs in antiviral immunity. Semin. Cell Dev. Biol..

[23] Fortes P., Morris K.V. (2016). Long noncoding RNAs in viral infections. Virus Res..

[24] Shi Q., Li Z., Dong Y., Yang G., Li M. (2023). LncRNA THRIL, transcriptionally activated by AP-1 and stabilized by METTL14-mediated m6A modification, accelerates LPS-evoked acute injury in alveolar epithelial cells. Int. Immunopharmacol..

[25] Rahni Z., Hosseini S.M., Shahrokh S., Saeedi Niasar M., Shoraka S., Mirjalali H., Nazemalhosseini-Mojarad E., Rostami-Nejad M., Malekpour H., Zali M.R. (2023). Long non-coding RNAs ANRIL, THRIL, and NEAT1 as potential circulating biomarkers of SARS-CoV-2 infection and disease severity. Virus Res..

[26] Carnero E., Barriocanal M., Segura V., Guruceaga E., Prior C., Borner K., Grimm D., Fortes P. (2014). Type I Interferon Regulates the Expression of Long Non-Coding RNAs. Front. Immunol..

[27] Xu J., Wang P., Li Z., Li Z., Han D., Wen M., Zhao Q., Zhang L., Ma Y., Liu W. (2021). IRF3-binding lncRNA-ISIR strengthens interferon production in viral infection and autoinflammation. Cell Rep..

[28] Pan Q., Zhao Z., Liao Y., Chiu S.H., Wang S., Chen B., Chen N., Chen Y., Chen J.L. (2019). Identification of an Interferon-Stimulated Long Noncoding RNA (LncRNA ISR) Involved in Regulation of Influenza A Virus Replication. Int. J. Mol. Sci..

[29] Ouyang J., Hu J., Chen J.L. (2016). lncRNAs regulate the innate immune response to viral infection. Wiley Interdiscip. Rev. RNA.

[30] Ouyang J., Zhu X., Chen Y., Wei H., Chen Q., Chi X., Qi B., Zhang L., Zhao Y., Gao G.F. (2014). NRAV, a long noncoding RNA, modulates antiviral responses through suppression of interferon-stimulated gene transcription. Cell Host Microbe.

[31] Lin H., Jiang M., Liu L., Yang Z., Ma Z., Liu S., Ma Y., Zhang L., Cao X. (2019). The long noncoding RNA Lnczc3h7a promotes a TRIM25-mediated RIG-I antiviral innate immune response. Nat. Immunol..

[32] Wang Y., Wang P., Zhang Y., Xu J., Li Z., Li Z., Zhou Z., Liu L., Cao X. (2020). Decreased Expression of the Host Long-Noncoding RNA-GM Facilitates Viral Escape by Inhibiting the Kinase activity TBK1 via S-glutathionylation. Immunity.

[33] Liu W., Wang Z., Liu L., Yang Z., Liu S., Ma Z., Liu Y., Ma Y., Zhang L., Zhang X. (2020). LncRNA Malat1 inhibition of TDP43 cleavage suppresses IRF3-initiated antiviral innate immunity. Proc. Natl. Acad. Sci. USA.

